# What makes health systems resilient against infectious disease outbreaks and natural hazards? Results from a scoping review

**DOI:** 10.1186/s12889-019-7707-z

**Published:** 2019-10-17

**Authors:** Jennifer B. Nuzzo, Diane Meyer, Michael Snyder, Sanjana J. Ravi, Ana Lapascu, Jon Souleles, Carolina I. Andrada, David Bishai

**Affiliations:** 1 Johns Hopkins Center for Health Security, 621 East Pratt Street, Suite 210, Baltimore, MD 21202 USA; 2 Johns Hopkins Bloomberg School of Public Health, 615 N. Wolfe Street, Baltimore, MD 21205 USA

**Keywords:** Resilience, Health system strengthening, Health system resilience, Quality improvement, Health security, Outbreak, Natural hazard

## Abstract

**Background:**

The 2014–2016 Ebola outbreak was a wake-up call regarding the critical importance of resilient health systems. Fragile health systems can become overwhelmed during public health crises, further exacerbating the human, economic, and political toll. Important work has been done to describe the general attributes of a health system resilient to these crises, and the next step will be to identify the specific capacities that health systems need to develop and maintain to achieve resiliency.

**Methods:**

We conducted a scoping review of the literature to identify recurring themes and capacities needed for health system resiliency to infectious disease outbreaks and natural hazards and any existing implementation frameworks that highlight these capacities. We also sought to identify the overlap of the identified themes and capacities with those highlighted in the World Health Organization’s Joint External Evaluation. Sources of evidence included PubMed, Web of Science, OAIster, and the websites of relevant major public health organizations.

**Results:**

We identified 16 themes of health system resilience, including: the need to develop plans for altered standards of care during emergencies, the need to develop plans for post-event recovery, and a commitment to quality improvement. Most of the literature described the general attributes of a resilient health system; no implementation frameworks were identified that could translate these elements into specific capacities that health system actors can employ to improve resilience to outbreaks and natural hazards in a variety of settings.

**Conclusions:**

An implementation-oriented health system resilience framework could help translate the important components of a health system identified in this review into specific capacities that actors in the health system could work to develop to improve resilience to public health crises. However, there remains a need to further refine the concept of resilience so that health systems can simultaneously achieve sustainable transformations in healthcare practice and health service delivery as well as improve their preparedness for emergencies.

## Background

Health system resilience has been previously defined as “the capacity of health actors, institutions, and populations to prepare for and effectively respond to crises; maintain core functions when a crisis hits; and, informed by lessons learnt during the crisis, reorganize if conditions require it” [1, 2]. For many countries, the 2014–2016 Ebola outbreak in West Africa was a wake-up call regarding the critical importance of having resilient health systems. In each of the three countries most affected by Ebola, a fragile health system was quickly overwhelmed by the complexity of tracking cases, the need to create and disseminate communication strategies, and the challenges of safely caring for a surge of critically ill patients. Health workers were 21–32 times more likely to have been infected with the virus than members of the general public [3]. Sickened health workers could no longer care for Ebola patients, and poor infection control in healthcare facilities contributed to nosocomial Ebola transmission. In turn, heightened risks of nosocomial Ebola infection increased public fear around hospitalization [4]. Rather than helping to contain Ebola, health systems became an amplifier of disease, exacerbating the human, economic, and political toll of the outbreak.

Similarly, unprepared health systems across the world inadvertently contributed to disease transmission during recent epidemics of Severe Acute Respiratory Syndrome (SARS) and Middle East Respiratory Syndrome (MERS) [5]. Health systems that were unprepared for disasters were also unable to provide essential services, even in highly developed countries (e.g., Canada during SARS [6], Korea with MERS [7], and the US following Hurricane Sandy [8]). Many countries have committed resources and efforts toward health system strengthening based on these recent disasters, but actionable plans and approaches to build resilient health systems have not yet achieved consensus.

Independent reviews of the global response to the 2014–2016 Ebola outbreak have stressed the importance of establishing metrics to assess and monitor progress towards improving countries’ capacity to respond to public health emergencies [9–11]. In 2016, the World Health Organization (WHO) created the International Health Regulations (IHR) Joint External Evaluation (JEE) tool—a framework and process designed to measure countries’ capacities to implement the requirements of the IHR, which include the ability to prevent, detect, and respond to public health emergencies of international concern [12]. Since its introduction, the JEE has become an important tool used by countries to assess their capacities for infectious disease outbreaks and other public health emergencies. To-date, more than 100 countries have conducted JEE assessments [13]. Some countries that have undergone JEE assessments have also begun to develop action plans to address gaps found in their JEEs. Despite this progress, health facilities continue to be vulnerable to public health emergencies [14].

Important work has been done to describe the general attributes of a resilient health system [1, 2, 15–17]. For example, Kruk et al. describe a resilient health system as one that is “integrated with existing efforts to strengthen health systems,” able to “detect and interpret local warning signs and quickly call for support,” able to provide care for a diverse population, able to “isolate threats and maintain core functions,” and is able to “adapt to health shocks” [2]. However, as highlighted by Turenne et al., there continues to be a lack of clarity around the conceptualization of health systems resilience [18].

The aim of this scoping review was to draw from existing literature to characterize specific capacities required to build resilient health systems in the face of infectious disease emergencies and natural hazards, with an emphasis on highlighting potential efforts that health system actors (e.g. health facilities and health service delivery organizations that are not always well-integrated in government-led preparedness initiatives) could pursue to achieve desired health outcomes during health crises. We also sought to examine the extent to which capacities that are associated with resilient health systems are addressed by existing frameworks for measuring and motivating countries’ health security, such as the JEE.

## Methods

We searched the scholarly and grey-literature databases to identify which capacities should be included in a framework for assessing and improving health system resilience to infectious disease outbreaks and natural hazards. We also sought to determine whether there were existing frameworks that highlighted these capacities that could be used in low-, middle-, and high-income settings. For the purposes of our research, we used the WHO definition of health systems, defined as “all the activities whose primary purpose is to promote, restore, or maintain health” [19]. Specifically, we integrated literature in the following three areas: health security, health systems strengthening, and quality improvement. The aims of this research were to characterize the impacts that infectious disease outbreaks and natural hazards have on health systems; to identify challenges in maintaining health service delivery during outbreaks and natural hazards; and to identify strategies for effecting sustainable change in health systems-strengthening efforts.

Literature databases included PubMed, Web of Science, and OAIster. Key search terms were informed by, but not inclusive of, Kruk et al.’s definition of a resilient health system, and included “health system,” “health system strengthening,” “resilience,” “recover,” “quality improvement,” “infectious disease,” “outbreak,” “natural disaster,” “global health security,” “pandemic,” “outbreak response,” and “essential functions,” as well as a variety of different pathogens responsible for recent infectious disease outbreaks (e.g., SARS, Ebola) and natural hazard types (e.g., cyclone, earthquake).^1^ See Additional file 1: Table S1 for the full electronic search strategy. Additionally, we examined the websites of major relevant public health organizations (WHO, the Rockefeller Foundation, CDC Stacks) to identify articles and frameworks not indexed in the aforementioned databases.

All but one of the search results were filtered to include only those articles published during or after 2002, to capture literature emanating in the wake of the SARS epidemic, up until February 2018, the end of the study period. However, one search term did included articles published during or after 1990, to capture more broadly those resources that focused on essential health functions. Only English-language articles were considered. We included documents if they described health system capacities that could potentially strengthen health system resilience to either infectious disease threats or natural hazards. Documents were excluded if they described health capacities that were outside the aims of this research, as defined previously (i.e. articles that were purely about public health capacities that did not mention the relationship of these capacities to the healthcare system). For example, articles that described the importance of a trained epidemiologic workforce (a public health capacity) in outbreak identification and mitigation would be excluded. Articles about the importance of engagement between Ministries of Health and the public would be excluded; however, articles about the importance of engagement between healthcare facilities and Ministries of Health would be included. Documents were also excluded if the article described resilience in contexts outside of natural hazards and infectious disease outbreaks (i.e. armed conflict situations).

Each of the research team members (7 in total) was assigned a set of articles to review. Each article title was reviewed by the assigned researcher for relevancy using the previously mentioned inclusion and exclusion criteria, followed by a review of the abstract for those titles deemed relevant. All articles deemed relevant after title and abstract review were then read in their entirety by the assigned researcher, providing a final set of articles for analysis. Article references were also reviewed to identify important literature not located in the primary search. Articles were then thematically coded by the assigned researcher using QRS International’s NVivo 11 coding software [20] and a qualitative coding instrument developed from a priori themes previously identified in other resilience checklists [1, 2, 21, 22]. Additional topics of interest that did not fit into the previously identified thematic rubric were coded as “other” for further review during data analysis. After completion of coding, through a process of inductive and deductive reasoning, the researchers identified a final list of themes and associated key literature that described the critical capacities necessary for health system resilience to infectious disease outbreaks and natural hazards. We then sought to identify areas of overlap between the health system resilience themes and capacities identified in our literature search, and the specific health security capacities that are the focus of the JEE.

## Results

The search yielded a total of 1108 articles after the removal of duplicates (Additional file 3: Figure S1). One hundred and fifty-eight articles were read in their entirety, of which 132 were deemed to be relevant and underwent thematic coding. After the completion of coding, we identified 77 key documents that described 16 high-level themes of health system resilience, which are summarized in Additional file 1: Table S2 (see Additional file 2: Appendix A and Appendix B for a comprehensive breakdown of sources organized by theme and author). Thirty-nine papers focused primarily on infectious diseases, while another 12 addressed natural hazards. The remaining 26 papers were not threat-specific, but rather articulated general principles for strengthening health systems and described baseline capacities required for health system functioning. While the themes found in our search were consistent with the five elements of a resilient health system previously outlined by Kruk et al. [1, 2], we also identified three additional themes not included in previous reviews, including the need to:

- Develop policies for determining what level of care will be delivered when the level of demand exceeds existing resources;
- Plan for post-event recovery; and
- Commitment to quality improvement that ensures integration of lessons learned.

For example, Mehta et al. described the need to develop “altered standards of care” for responses to mass casualty events, which might include shifting resources to save as many lives as possible (i.e., triaging patients differently during emergencies as compared to normal operating conditions) and allowing for group isolation of patients that would normally be boarded in single rooms [23]. The literature identified a number of issues that must be addressed during the recovery phase of a public health emergency, including the need for grief and psychological counseling [24], after-action assessment and revision of emergency response plans [25], and rebuilding of social cohesion and trust [26]. A commitment to continuous quality improvement was also identified as an important component of resilient health systems, including making hospital performance ratings mandatory and publicly available to encourage peer competition with the primary goal being the overall improvement of hospital performance [27].

In integrating literature across subject areas, we were able to identify multiple references to the capacities necessary to achieve the 16 health system resilience attributes identified in our scoping review, which are summarized below (also see Additional file 1: Table S2).

- Core Health Service Capabilities: A resilient health system sustains baseline levels of routine healthcare delivery during a public health emergency [28–35].
- Barriers to Healthcare Access: A resilient health system dismantles barriers to healthcare access so that the public accesses care during emergencies [36, 37].
- Maintaining Critical Infrastructure and Transportation: A resilient health system develops plans to weather interruptions in critical infrastructure and transportation [24, 25, 38–50].
- Timely and Flexible Access to Emergency/Crisis Financing: A resilient health system has timely, flexible access to financing so that it can better prepare for and respond to public health emergencies [24, 29, 36, 37, 39–41, 51–59].
- Leadership and Command Structure: A resilient health system has a clear and flexible command structure that has been established prior to an event and is exercised frequently [24, 29, 57, 60–65].
- Collaboration, Coordination, and Partnerships: A resilient health system collaborates and coordinates with partners within and outside of the health system [2, 29, 39, 45, 57, 66, 67].
- Communication: A resilient health system has clear channels of communication between health system actors and other sectors, risk communication protocols, and robust engagement with patient populations [60, 68].
- Flexible Plans and Management Structures: A resilient health system has flexible plans and management structures to cope with rapidly evolving circumstances [1, 69–71].
- Legal Preparations: A resilient health system has made legal preparations to address challenges that may emerge during a crisis [1, 45, 49, 59, 62, 72, 73].
- Surge Capacity: A resilient health system is able to call on human and capital resources to “surge” the level of care during public health emergencies [36, 74].
- Altered Standards of Care: A resilient health system has adaptable response plans to guide them in allocating scarce resources and healthcare services [23, 75].
- Health Workforce: A resilient health system has an adequate, trained, and willing work force [4, 22, 36, 40, 45, 53, 54, 76, 77].
- Medical Supplies and Equipment: A resilient health system has access to medical supplies and equipment, including personal protective equipment, antivirals, and ventilators, during a crisis [40, 53, 78–80].
- Infection Prevention and Control (IPC): A resilient health system has implemented strong IPC measures, including staff training, standardized protocols, a dedicated IPC focal point, and dedicated treatment units [4, 22, 39, 47, 51, 54, 81–86].
- Commitment to Quality Improvement: A resilient health system requires a commitment to continuous quality improvement that promotes excellence and garners the trust of the community [15, 27, 39, 54, 69, 87, 88].
- Plans for Post-Event Recovery: Resilient health systems have plans for post-event recovery that address a broad range of issues [24–26, 34, 40, 59, 73, 89–93].

The capacities that we identified are associated with different actors in health systems. Some of the capacities identified in our review could potentially be developed by individual health facilities. For example, Kim et al. discussed one health system’s plan to develop alternate care centers that could be deployed during an influenza pandemic, including the infrastructure that needs to be in place to ensure adequate functioning, such as transport of patients to the center [42]. Other capacities identified in the scoping review concerned the health system more broadly and would likely be addressed by national governmental authorities. For example, Hanefeld et al. noted “the nature of the funding and financing mechanism as a core aspect enabling or hindering health systems’ ability to respond to a shock” [29].

No frameworks were identified in the search that translated these high-level themes into specific and actionable steps that health system actors can employ to improve and support health system resilience to both infectious diseases and natural hazards, and that can be undertaken in low-, middle-, and high-income settings alike. Frameworks that did articulate specific capacities were either 1) only applicable in the US context [22] or 2) did not cohesively address both infectious diseases and natural disasters [50]. For example, Meyer et al. created a checklist for health sector resilience to high-consequence infectious diseases [22], but the data for this checklist was informed by the US domestic response to the 2014–2016 West Africa Ebola outbreak. While some of the identified capacities may be generalizable to other countries, some are only pertinent in the US context. Similarly, the Hospital Safety Index, a tool developed by WHO, does identify capacities that are relevant to some health facilities, but the tool is largely aimed at evaluating the vulnerability of hospital infrastructure to natural hazards (an updated version includes limited consideration of the potential impact of infectious disease threats to hospitals) [50].

Only two of the health system resilience themes and capacities identified in our literature search directly overlapped with the specific health security capacities that are the focus of the JEE—namely IPC and communication (See Additional file 1: Table S3). Specifically, the JEE indicator on antimicrobial resistance does address IPC, but only within the context of healthcare-associated infections and associated IPC programs [12]. Additionally, the literature we collected emphasized the importance of communication between health system actors, other sectors, and the community during outbreaks [60, 68]. The JEE contains a very detailed section on communication that specifically calls out for the need for communication and coordination between stakeholders, including healthcare workers; for systems for rumor management through healthcare workers; and for formal communication mechanisms with the hospital and healthcare sector [12].

Otherwise, health facilities are not directly addressed in the JEE framework. There are some indicators in the JEE that do address the themes identified in the literature review, but only within the context of public health. For example, the literature suggests that health facilities need access to financing during emergencies to cover the added costs of preparing for and responding to emergencies [24, 29, 36, 37, 39–41, 51–59]. The JEE indicator on National, Legislation, Policy and Financing does address whether countries have the financing to fulfill their obligations under the IHR, which includes “regulations or administrative requirements, or other governmental instruments governing public health surveillance and response” [12]. However, it does not specifically address financing within the context of health facilities, although countries could choose to include them in efforts to develop capacities in those areas.

Finally, some of the themes identified in our review could be leveraged through the development of other capacities that are the focus of the JEE. For example, the JEE does not explicitly assess how healthcare facilities should address barriers to healthcare access, such as long travel distances, the high cost of medical care, and public distrust. However, it does address the importance of risk communication and community engagement during an emergency. These relationships could potentially be leveraged by the healthcare system during an emergency to improve the public’s trust in and subsequent use of the healthcare system.

## Discussion

To date, much of the literature that specifically references health system resilience has focused on high-level attributes, rather than identifying specific capacities that health systems need to be resilient to infectious disease outbreaks and natural hazards. For example, Kruk et al.’s five attributes of a resilient health system include a system that is “self-regulating,” with the ability to “quickly identify and isolate a threat and target resources to it” [1]. By integrating literature from across different disciplines, we were able translate these high-level themes into actionable corresponding capacities that health systems need to respond to infectious disease outbreaks and natural hazards. For example, the literature highlighted numerous IPC protocols and practices that are important for the control of infectious disease threats, including the need for front-line healthcare workers to conduct travel histories [22] and the need to establish dedicated and multidisciplinary IPC committees to coordinate and guide healthcare staff on how to safely manage patients with infectious diseases [54, 84]. An article by Palagyi et al., published after this review was conducted, also highlights the importance of these capacities that we identified [94].

Additionally, the literature highlighted three themes not previously identified as attributes of a resilient health system, which warrant consideration in future efforts to define health system resilience. We present the capacities that we identified across the literature as merely the beginning of an effort to define capacities that health system actors need to be prepared for infectious disease outbreaks and natural hazards. Further scholarship in these areas could support efforts to translate research findings into best practices in public health and healthcare practice and improve health outcomes following public health emergencies of all kinds.

Notably, the JEE does identify the capacities necessary to implement the IHRs to protect against public health emergencies of international concern, but lacks guidance for health facilities at the patient-provider interface [12]. Moreover, many of the capacities assessed in the JEE presume the existence and functioning of core health system capacities, yet these capacities themselves are not explicitly addressed in JEE assessments. For example, while the presence of a national laboratory system—a JEE indicator—is a critical capacity to have during an outbreak, it requires that healthcare providers and the proper supplies be available to collect patient samples (e.g., blood, sputum, etc.). Ideally, efforts to improve health system resilience would complement and build upon those foundational capacities presumed by the JEE process.

The results of this literature review point to a need for increased integration of efforts to advance health security and health systems strengthening across the globe. Several high-priority elements for health systems resilience likely exist at the nexus of health systems strengthening, health system resilience, and health security; further work is needed to determine the most effective co-investments in global health security and health system strengthening that enable more robust health system responses at the local, national, regional, and global levels during emergent crises [95]. Identifying those areas of overlap can help to actualize the JEE’s priority areas in health security, and also strengthen key components of national health systems such that their overall resilience is enhanced.

While we strived to capture all relevant health systems literature, a potential limitation to our review is the lack of consistency and definitional clarity with which terms like “health system” and “resilience” are used throughout the medical and public health literature. It is possible that we may have missed relevant articles that describe these concepts using different terms. We also found in the literature an overrepresentation of papers detailing health system impacts of certain events and, thus, our findings may not include considerations from other events not represented in the literature. Despite these limitations, we think our review serves to deepen the understanding of the specific capacities that health systems need to prepare for infectious diseases and natural hazards.

## Conclusions

The themes and capacities identified in our literature review provide an initial step in refining the concept of health system resilience to enable actors across the various sectors of the health system to take action to be able to respond and recover from infectious disease outbreaks and natural hazards. There remains a need to further define the concept of resilience so that health systems can simultaneously achieve sustainable transformations in public health practice and health service delivery as well as improve their preparedness for emergencies. In the same way that the JEE tool has helped motivate countries to assess and improve their core public health capacities, an implementation-oriented health system resilience framework could help translate the important components of a health system identified in this review into specific capacities that actors in the health system could work to develop to improve resilience to public health crises. Moreover, such an effort may help to integrate foundational health system capacities into national efforts to improve core public health capacities.

## Supplementary information

**Additional file 1:** **Table S1.** Electronic search strategy. **Table S2.** Summary of key themes and associated key evidence. **Table S3.** Overlap of scoping review themes with Joint External Evaluation indicators.

**Additional file 2:** Appendix A: Coding framework--the full coding framework is provided, along with definitions of each code, and the number and first author/publication year of all sources that were coded into each theme. Appendix B: Citations are provided for all sources referenced in appendix A.

**Additional file 3:** **Figure S1.** Selection of Sources of Evidence.

## Data Availability

not applicable.

## Abbreviations

IPC: Infection Prevention and Control
JEE: Joint External Evaluation
MERS: Middle East Respiratory Syndrome
SARS: Severe Acute Respiratory Syndrome
US: United States
WHO: World Health Organization

## Footnotes

1 Kruk et al.’s definition of a resilient health system was used to inform the search terms because it was developed with broader expert input and because it addresses resilience within the context of infectious disease outbreaks and natural hazards.
