# Supplementary material for: What makes health systems resilient against infectious disease outbreaks and natural hazards? Results from a scoping review
Source: BMC Public Health. 2019 Oct 17;19:1310. doi: 10.1186/s12889-019-7707-z (PMC6798426; doi:10.1186/s12889-019-7707-z)
Supplement: Supplementary file 1 — Additional file 1: Table S1. Electronic search strategy. Table S2. Summary of key themes and associated key evidence. Table S3. Overlap of scoping review themes with Joint External Evaluation indicators. [file 12889_2019_7707_MOESM1_ESM.docx]

Table S1: Electronic search strategy

| - “health system” AND resilience AND (outbreak OR “natural disaster”) - “health system” AND recovery AND (outbreak OR “natural disaster”) - “health system” AND “quality improvement” AND (outbreak OR “natural disaster”) - “health system” AND (SARS OR Ebola OR MERS OR plague OR nipah OR cyclone OR hurricane OR tsunami OR earthquake OR flooding OR tornado) - “health system strengthening” AND (“infectious disease” OR outbreak OR “natural disaster” OR “global health security”) - resilience AND (pandemic OR Ebola OR MERS OR SARS OR plague OR nipah OR outbreak OR “outbreak response”) - “health system” AND “essential functions” AND (outbreak OR “natural disaster”)* |
| --- |

*Filtered for publication date 01/1990-02/2018. All other terms filtered for publication date after 01/2002-02/2018

Table S2: Summary of themes and associated key evidence

| **Theme** | **Summary of Key Evidence** | **Key Evidence*** |
| --- | --- | --- |
| Core Health Service Capabilities | The ability to sustain baseline levels of routine healthcare delivery during a public health emergency is a hallmark of health system resilience. These baseline services might include physical therapy and rehabilitation, internal medicine, essential surgery and trauma, maternal/fetal care, and pediatric medicine. Harmonizing vertical public health and healthcare programs with horizontal health system-strengthening efforts prior to emergencies improves the provision of both baseline and emergency health services. | Elliott (2011), Hanefeld (2018), Lapão (2015), Lohman (2016), Loubet (2015), Orenstein (2016), Reinhardt (2011), Siekmans (2017) |
| Barriers to Healthcare Access | Dismantling barriers to healthcare access—including high costs of care, long travel distances, asymmetric distributions of healthcare resources, and public mistrust of health institutions—strengthens resilience by increasing health service utilization during emergencies, creating opportunities to prevent and detect cases at the patient-provider interface. Greater integration of emergency services into existing health systems, including services for vulnerable populations (eg, refugees), enables more responsive care. | Ammar (2016), Liu (2004) |
| Maintaining Critical Infrastructure and Transportation | Health systems must develop plans to weather interruptions in critical infrastructure and transportation, such as back-up systems or contingencies to transfer patients to alternate care sites. Examples include interruptions in electricity or other power sources, food, water, telecommunications, sewage and waste management, information management, and transportation. In some environments, long-standing gaps in infrastructure at health facilities that compromise routine care could also exacerbate the tolls inflicted by an emergency. | Ardagh (2012), Cancedda (2016), Elston (2017), Pan American Health Organization (2008), Jones (2017), Kearns (2014), Kim (2009), Kingham (2009), Moore (2015); Pouraghaei (2017), Rozeman (2006), Saghafi Nia (2008), Shoman (2017), Tran (2015), Yakubu (2014) |
| Timely and Flexible Access to Emergency/Crisis Financing | Health systems that have timely, flexible access to financing can better prepare for and respond to public health emergencies. External shocks pose significant resource and financial costs on health systems, ranging from higher expenditures for medical supplies to additional staffing demands. Insufficient funding in advance of a crisis hinders the ability of health facilities and government to appropriately plan, prepare, and respond to an emergency. | Ammar (2016), Cancedda (2016), DeBiasi (2016), Elmahdawy (2017), Elston (2017), Felland (2008), Hanefeld (2018), Harries (2010), Ivers (2011), Jones (2017), Karanikolos (2016), Kearns (2014), Liu (2004), Van Minh (2014), Wurie (2016), Zhong (2014) |
| Leadership and Command Structure | Skilled, flexible leadership and clear command structures are essential for responding to public health emergencies. These structures should be put into place prior to an event and exercised frequently to enable clear channels of communication and familiarize individuals with their designated roles. Incident management systems, including emergency operation centers, should include empowered representatives from the health system, as well as community stakeholders. | Cleary (2010), Dhanoa (2014), Elston (2017), Hanefeld (2018), McMahon (2017), Regmi (2015), Van Minh (2014), Varma (2017), Yantao (2011) |
| Collaboration, Coordination, and Partnerships | Collaboration, coordination, and partnerships, both within the health system and between other sectors, enhance resilience by improving emergency response time and access to financial, intellectual, and human resources. Examples include partnerships with international, national, and local government agencies, emergency medical services, and civil society and NGOs. This should not overlook the involvement of the greater community in preparedness, planning, and implementation activities, including schools, local employers, and faith-based organizations. | Cancedda (2016), Chi (2015), Hanefeld (2018), Kruk (2017), Ling (2017), Lurie (2004), Rozeman (2006), Van Minh (2014) |
| Communication | Strong communication capabilities – including channels of communication between health system actors and sectors, risk communication protocols, and robust engagement with patient populations and communities affected by the crisis in question – are critical enabling factors of efforts to build resilient health systems. Community engagement strategies preserve public confidence in the health system and promote utilization of health services. | Beard (2003), Cleary (2010) |
| Flexible Plans and Management Structures | Resilient organizations tend to have flexible plans and management structures, enabling them to cope with rapidly evolving circumstances and changes in patient expectations and demands. System-wide flexibility must be complemented by a more granular understanding of how individuals’ roles and actions may change during crisis (e.g., reprioritization of clinical activities by health workers as a crisis evolves). | Barasa (2017), Gizelis (2017), Kruk (2015), Martineau (2016) |
| Legal Preparations | Unresolved legal questions can hamper health systems’ abilities to respond to emergencies. Legal preparation includes legislation authorizing agencies to respond to an emergency, as well as legal guidance determining the scope of responsibility among different agencies to enable more efficient coordination of response efforts. | Campbell (2004), Kruk (2015), McMahon (2017), Rozeman (2006), Verni (2012), Yakubu (2014), Zhong (2014) |
| Surge Capacity | Health service organizations must be able to call on human and capital resources to “surge” the level of care during public health emergencies. This includes reserves of adequately trained personnel; adequate medical supplies, equipment, and available beds; as well as policies and procedures to provide guidance during the emergency. | Ammar (2016), Therrien (2017) |
| Altered Standards of Care | Health systems should be prepared to deliver altered standards of care during crises. These should be reflected in adaptable response plans to guide health systems in allocating scarce resources and healthcare services, thereby ensuring the best outcomes for the highest number of patients. | Institute of Medicine (2012), Mehta (2006) |
| Health Workforce | The availability of an adequate, trained, and willing work force is a critical component of health system resilience. Occupational health programs and regularly scheduled drills and exercises keep the workforce healthy and prepared. Hospitals should be prepared to provide food, space, counseling/psychosocial support and other resources to hospital staff. Preparedness plans to mobilize volunteers mitigate workforce shortages during an emergency. | Ammar (2016), Felland (2008), Gostin (2015), Harries (2010), Jones (2017), Lewis (2017), Meyer (2017), Raven (2018), Rozeman (2006) |
| Medical Supplies and Equipment | Health systems must ensure continued access to medical supplies and equipment, including personal protective equipment, antivirals, and ventilators. If facilities are unable to provide access to these resources, then plans should be in place for patient referral and transport to facilities that do have access. | Felland (2008), Jones (2017), Rebmann (2017), Reece (2017), Schwanke Khilji (2013) |
| Infection Prevention and Control | The provision of adequate levels of infection control is essential during outbreaks. This includes staff training and guidance on proper infection prevention and control measures; standardized, routine protocols for the screening, isolation, care and transport of patients with highly infectious diseases; a dedicated focal point in which questions about infection control can be directed; and dedicated units for the treatment of infectious patients. | Barden-O’Fallon (2015), Cancedda (2016), Cooper (2016), DeBiasi (2016), Elston (2016), Gostin (2015), Harries (2010), Meyer (2017), Pathmanathan (2014), Shoman (2017), Singh (2017), Subhash (2016) |
| Commitment to Quality Improvement | A resilient health system requires a commitment to continuous quality improvement that promotes excellence and garners the trust of the community. Transparency, such as accreditation and public hospital performance ratings, can promote quality improvement and strengthen public trust by widening the circle of community members who are stewards of the health system. | Barasa (2017), Blanchet (2017), Cancedda (2016), Hafner (2017), Harries (2010), Shibuya (2011), World Health Organization (2017) |
| Plans for Post-Event Recovery | Resilient health systems have plans for post-event recovery that address a broad range of issues. These might include grief and psychological counseling; navigating food shortages and price increases; providing immunizations; education and employment opportunities for survivors, widows, and orphans; and rebuilding social cohesion and trust. A framework or systematic approach helps to ensure these diverse recovery activities are organized effectively. | World Health Organization (2016), Elston (2017), Fitter (2017), Jones (2017), McPake (2015), Moore (2015), Oliveira (2015), Reinhardt (2011), Shultz (2016), Tambo (2017), Verni (2012), Zhong (2014) |

*For full citations see Appendix B

Table S3: Overlap of Scoping Review Themes with Joint External Evaluation (JEE) Indicators

| **Literature Review Theme** | **Comparable JEE Indicator(s)** | **Rationale** |
| --- | --- | --- |
| Core Health System Capabilities^1^ | No comparable indicator | While the JEE addresses numerous core capabilities and capacities that are not unrelated to healthcare, including immunization, laboratory systems, surveillance, and reporting, it does not specifically call out the need to maintain these capacities within the healthcare system. Additionally, it does not mention the need to maintain access to core healthcare services during emergencies, such as maternal/fetal care, internal medicine, and rehabilitation and physical therapy services. |
| Barriers to Healthcare Access^2^ | Risk Communication | The JEE does not explicitly assess how healthcare facilities should address barriers to healthcare access, such as long travel distances, the high cost of medical care, and public distrust. However, it does address the importance of risk communication and community engagement during an emergency (see R.5.3 Public Communication; R.5.4 Communication Engagement with Affected Communities). These relationships could potentially be leveraged by the healthcare system during an emergency to improve the public’s trust in and subsequent use of the healthcare system. |
| Maintaining Critical Infrastructure and Transportation^2^ | Biosafety and Biosecurity | JEE indicator question P.6.2 assesses the presence of biosafety and biosecurity training and practices that ensure the safe management of especially dangerous pathogens, including waste management policies. The indicator on radiation emergencies (RE.2) mentions the transport of radioactive waste. However, waste management policies specific to the healthcare system and infectious diseases are not noted. Additionally, there is no explicit mention of the need to maintain and secure critical infrastructure and transportation during emergencies. |
| Timely and Flexible Access to Financing^3^ | National Legislation, Policy and Financing | JEE indicator question P.1.1 includes an assessment of whether countries have the financing in place to fulfill their obligations under the International Health Regulations, which includes “regulations or administrative requirements, or other governmental instruments governing public health surveillance and response.” However, access to financing for health facilities during a response is not explicitly mentioned. |
| Leadership and Command Structure^3^ | Preparedness, Emergency Response Operations | The JEE includes an assessment of whether countries have emergency preparedness and response plans (see R.1.1- “Multi-hazard national public health emergency preparedness and response plan is developed and implemented”), including whether these plans are exercised and updated. Additionally, question R.2.2 addresses EOC Operating Procedures and Plans, which should include a description of the incident management structure. However, it does not explicitly mention these in the context of individual healthcare facilities. |
| Collaboration, Coordination, and Partnerships^3^ | IHR Coordination, Communication and Advocacy | JEE indicator question P.2.1 assesses whether a country has mechanisms “established for the coordination and integration of relevant sectors in the implementation of IHR, to include those that might be relevant during a public health crisis.” While healthcare facilities would likely be considered a relevant sector, it is not explicitly called out. |
| Communication^4^ | IHR Coordination, Communication and Advocacy  Risk Communication | The JEE addresses communication between sectors (see P.2.1 described above) and partners (see R.5.2 Internal and Partner Communication and Coordination) and with the public (see R.5.3 Public Communication; R.5.5 Dynamic Listening and Rumour Management). It specifically calls out the need for communication and coordination between stakeholders, including the health care sector. |
| Flexible Plans and Management Structures^3^ | Preparedness, Emergency Response Operations | JEE indicator R.1.1 includes an assessment of whether countries have emergency preparedness and response plans. Additionally, the Emergency Response Operations indicator states that plans for emergency operations “should be developed that can be scalable and flexible to address emerging disease threats.” However, these are not mentioned explicitly within the context of healthcare facilities. |
| Legal Preparations^3^ | National Legislation, Policy and Financing | JEE indicator question P.1.1 (see above) includes an assessment of whether countries have the legal framework in place to fulfill their obligations under the International Health Regulations, which includes those “governing public health surveillance and response.” However, it does not explicitly state that legal preparations should also be made within the healthcare facility context. |
| Surge Capacity^3^ | Preparedness, Emergency Response Operations | JEE indicator question R.1.1 (see above) measures a country’s preparedness to respond to a public health emergency, including the existence of adequate surge capacity. Additionally, JEE indicator question R.2.2 assesses a country’s ability to stand up and maintain a public health emergency operation center during a public health emergency, including emergency response plans and identification of surge staff. However, surge capacity in the context of individual healthcare facilities is not addressed. |
| Altered Standards of Care^1^ | No comparable indicator |  |
| Healthcare Workforce^3^ | Workforce Development  Medical Countermeasures and Personnel Deployment | JEE indicator question D.4.1 assesses whether “human resources are available to implement IHR core capacity requirements,” including the presence of clinicians and nurses. Indicator question D.4.3 addresses workforce strategy, including workforce strategies for doctors and nurses. Indicator question R.4.2 assesses whether a system is in place for “sending and receiving health personnel during a public health emergency.” However, these are all discussed in the context of public health, and not individual healthcare facilities. |
| Medical Supplies and Equipment^3^ | Medical Countermeasures and Personnel Deployment | JEE indicator question R.4.1 addresses whether a system is in place for sending and receiving medical countermeasures during a public health emergency, such as antibiotics, vaccines, and equipment. However, ensuring adequate medical supplies and equipment within healthcare facilities is not explicitly addressed. |
| Infection Prevention and Control^4^ | Antimicrobial Resistance | JEE indicator P.3.3 addresses healthcare associated infection prevention and control (IPC) programs, including IPC policies, guidelines, and training for healthcare workers and the availability of isolation units. |
| Commitment to Quality Improvement^1^ | No comparable indicator |  |
| Plans for Post-Event Recovery^1^ | No comparable indicator |  |

1=no overlap

2=some capacities/capabilities identified in JEE could be leveraged by healthcare facilities

3=similar capacity/capability identified in JEE but healthcare facilities are not specifically mentioned

4=capacity/capability in healthcare facility is identified in JEE
