# Supplementary material for: What makes health systems resilient against infectious disease outbreaks and natural hazards? Results from a scoping review
Source: BMC Public Health. 2019 Oct 17;19:1310. doi: 10.1186/s12889-019-7707-z (PMC6798426; doi:10.1186/s12889-019-7707-z)
Supplement: Supplementary file 2 — Additional file 2: Appendix A: Coding framework--the full coding framework is provided, along with definitions of each code, and the number and first author/publication year of all sources that were coded into each theme. Appendix B: Citations are provided for all sources referenced in appendix A. [file 12889_2019_7707_MOESM2_ESM.docx]

**Appendix A: Coding Framework**

| **Code** | **Definition** | **Number of Sources** | **Sources** |
| --- | --- | --- | --- |
| Access and barriers to care | Barriers that exist that might prevent individuals from accessing care routinely and during public health emergencies. | 21 | Ammar (2016), Blanchet (2017), Dhariwal (2004), Elson (2017), Gostin (2015), Hanefeld (2018), Ivers (2011), Kao (2017), Kreisberg (2016), Kruk (2017), Ling (2017), Liu (2004), Lurie (2004), Macintyre (1999), McMahon (2017), Oliveria (2015), Omoleke (2016), Saghafi Nia (2008), Siekmans (2017), Veneema (2017), World Bank (2014) |
| Access to medical supplies, equipment, and medications | Systems or efforts to ensure continued access to medical supplies, equipment, and medications during public health emergencies. | 24 | Balfour (2016), Cancedda (2016), Elmahdawy (2017), Elston (2017), Felland (2008), Gauld (2005), Hafner (2016), Jones (2017), Schwanke Khilji (2013), Kluge (2017), Lapão (2015), Mehta (2006), Van Minh (2014), Moore (2015), Omoleke (2016), Rebmann (2017), Reece (2017), Rozeman (2006), Senga (2016), Simonetti (2017), Therrien (2016), Tran (2015), Verni (2012), Yakubu (2016) |
| Collaboration, coordination, and partnerships | Relationships that exist before and during a public health emergency that might improve resilience. | 19 | Ardagh (2012), Balfour (2016), Campbell (2004), Chaple (2017), DeBiasi (2015), Dhanoa (2017), Elston (2017), Garibaldi (2016), Ivers (2011), Kluge (2017), Van Minh (2014), Olu (2017), Olu, et al. (2017), Peters (2017), Read (2015), Saghafi Nia (2008), Schenk (2018), World Health Organization (2017), Varma (2017) |
| Collaboration, coordination, and partnerships: across health system and other sectors | Relationships that exist across the health system and other sectors that might improve resilience during a public health emergency. | 24 | Akbari (2005), Beard (2003), Blanchet (2017), Campbell (2004), Cancedda (2016), Chi (2015), Felland (2008), Hanefeld (2018), Jones (2017), Kearns (2014), Schwanke Khilji (2013), Kruk (2015), Kruk (2017), Lapão (2015), Ling (2017), Lurie (2004), McMahon (2017), McPake (2015), Mutale (2016), Rockefeller (2016), Rozeman (2006), Setiawan (2006), Therrien (2016), Verni (2012) |
| Collaboration, coordination, and partnerships: within the health system | Relationships that exist within the health system that might improve resilience during a public health emergency. | 23 | Ammar (2016), Ardalan (2009), Campbell (2004), Dhariwal (2004), Felland (2008), Gauld (2005), Gizelis (2017), Hanefeld (2018), Hartman (2012), Jones (2017), Kao (2017), Kennedy (2016), Kreisberg (2016), Kruk (2017), Ling (2017), Liu (2004), Oliveria (2015), Pouraghaei (2017), Setiawan (2006), Shamian (2007), Shoaf (2014), Verni (2012), World Bank (2014) |
| Core health system capabilities | The core health system capabilities needed to maintain resilience during a public health emergency, including maintaining normal functioning. | 24 | Ammar (2016), Andrus (2017), Dhariwal (2004), Elliott (2010), Elston (2015), Fitter (2017), Fukuma (2017), Griekspoor (2004), Harries (2010), Kennedy (2016), Schwanke Khilji (2013), Kreisberg (2016), Kruk (2015), Kruk (2017), Li (2016), Ling (2017), McMahon (2017), Meyer (2017), Orenstein (2016), Regmi (2015), Shibuya (2011), Shoaf (2014), Shoman (2017), World Bank (2014) |
| Crisis standards of care | Alterations in the standards of care that might be provided during a public health crisis. | 18 | Ardagh (2012), Balfour (2016), Bracha (2006), Campbell (2004), Cancedda (2016), DeBiasi (2015), Elston (2017), Institute of Medicine (2012), Schwanke Khilji (2013), Kreisberg (2016), Kruk (2017), Mehta (2006), Van Minh (2014), Pouraghaei (2017), Read (2015), Saghafi Nia (2008), Varma (2017), Yakuba (2016) |
| Critical infrastructure and transportation | The infrastructure and transportation that must be in place to ensure a resilient health system. | 37 | Akbari (2005), Ardagh (2012), Barasa (2017), Cancedda (2016), Chaple (2017), Cleary (2010), Cooper (2016), DeBiasi (2015), Dhariwal (2004), Elston (2015), Elston (2017), Felland (2008), Fitter (2017), Harries (2010), Pan American Health Organization (2008), Jones (2017), Kearns (2014), Kennedy (2016), Kim (2009), Kingham (2009), Kruk (2017), Macintyre (1999), Meyer (2017), Moore (2015), Omoleke (2016), Pouraghaei (2017), Rozeman (2006), Saghafi Nia (2008), Schenk (2017), Shoman (2017), Singh (2017), Tran (2015), Verni (2012), World Bank (2014), Wurie (2016), Yakubu (2016), Zhong (2014) |
| Financing | The presence of adequate resources to respond to public health crisis. | 32 | Ammar (2016), Cancedda (2016), DeBiasi (2015), Dhariwal (2004), Elmahdawy (2017), Elston (2017), Felland (2008), Hanefeld (2018), Harries (2010), Ivers (2011), Jones (2017), Kao (2017), Karanikolos (2016), Kearns (2014), Kennedy (2016), Kluge (2017), Kruk (2017), Ling (2017), Liu (2004), Macintyre (1999), Van Minh (2014), Olu (2017), Rebmann (2017), Schenk (2017), World Health Organization (2017), Shibuya (2011), Shoman (2017), Tomori (2015), Varma (2017), World Bank (2014), Wurie (2016), Zhong (2014) |
| Flexibility | The presence of flexible response plans and management structures during a public health crisis. | 13 | Ardagh (2012), Barasa (2017), Blanchet (2017), Fukuma (2017), Gizelis (2017), Hafner (2016), Institute of Medicine (2012), Kluge (2017), Kruk (2015), Kruk (2017), Ling (2017), Martineau (2016), World Health Organization (2017) |
| Infection control | The infection control processes and procedures needed to prevent spread of disease within hospitals. | 25 | Barden-O’Fallon (2015), Bracha (2006), Cancedda (2016), Hospitals prepare plans, (2014), Copper (2016), DeBiasi (2015), Gostin (2015), Harries (2010), Isakov (2015), Jones (2017), Loubet (2015), McMahon (2017), Meyer (2017), Pathmanathan (2014), Quaglio (2016), Saurabh (2017), Senga (2016), Sesay (2017), Shamian (2007), Shoman (2017), Singh (2017), Subhash (2016), Tomori (20150, Varma (2017), World Health Organization (2016) |
| Leadership and command structure | The leadership and command structures needed to respond to a public health crisis. | 31 | Ardagh (2012), Balfour (2016), Beard (2003), Campbell (2004), Chaple (2017), Hospitals prepare plans (2014), Cleary (2010), Dhanoa (2017), Elston (2017), Felland (2008), Gauld (2005), Hanefeld (2018), Kluge (2017), Kruk (2017), Liu (2004), Lohman (2016), McMahon (2017), McPake (2015), Nyenswah (2017), Regmi (2015), Rozeman (2006), Schenk (2017), Shoaf (2014), Shoman (2017), Tambo (2017), Varma (2017), Verni (2012), Wurie (2016), Yantao (2011), Zhang (2014), Zhong (2014) |
| Legal preparedness | The legal preparations needed to allow efficient response operations during a public health crisis. | 13 | Campbell (2004), Kluge (2017), Kruk (2015), Lurie (2004), McMahon (2017), Van Minh (2014), Nyenswah (2017), Olu (2017), Rozeman (2006), Varma (2017), Verni (2012), Yakuba (2016), Zhong (2014) |
| Maintaining baseline services | The capacities needed to ensure that baseline services are maintained during a public health crisis. | 31 | Ardagh (2012), Balfour (2016), Barasa (2017), Barden-O’Fallon (2015), Blanchet (2017), Cancedda (2016), Caulker (2017), DeBiasi (2015), Dhariwal (2004), Elliott (2010), Elston (2015), Elston (2017), Felland (2008), Gizelis (2017), Jones (2017), Jones (2016), Lapão (2015), Ling (2017), Lohman (2016), Loubet (2015), Oliveria (2015), Quaglio (2016), Reinhardt (2011), Rozeman (2006), Sesay (2017), Shannon (2017), Shoman (2017), Shultz (2017), Siekmans (2017), Therrien (2016), Zhong (2014) |
| Other | Other potential topics and themes identified that need further review. | 38 | Akbari (2005), Andrus (2017), Ardagh (2012), Baers (2018), Barasa (2017), Bolkan (2014), Bracha (2006), Campbell (2004), Cancedda (2016), Dhariwal (2004), Elmahdawy (2017), Fukuma (2017), Gauld (2005), Hafner (2016), Hanefeld (2018), Kao (2017), Kearns (2014), Kruk (2017), Lapão (2015), Li (2016), Lien (2014), Ling (2017), Lohman (2016), McPake (20150, Mehta (2006), Olu (2017), Omoleke (2016), Paudel (2016), Pouraghaei (2017), Reece (2017), Reinhardt (2011), Setiawan (2006), Shoaf (2014), Shoman (2017), Tambo (2017), Veneema (2017), Verni (2012), World Bank (2014) |
| Post-event recovery | The capacities needed to ensure post-event recovery. | 18 | Akbair (2005), Balfour (2016), Elston (2017), Fitter (2017), Jones (2017), McPake (2015), Van Minh (2014), Moore (2015), Mussah (2017), Oliveira (2015), Reinhardt (2011), Saghafi Nia (2008), Shultz (2017), Tambo (2017), USAID (2011), Verni (2012), World Bank (2014), Zhong (2014) |
| Quality improvement of the health system | The systems needed to improve the quality of the health system to ensure resilience. | 11 | Barasa (2017), Blanchet (2017), Cancedda (2016), Hafner (2016), Harries (2010), Khiji (2013), Mutale (2017), Oliveira (2015), Peters (2017), World Health Organization (2017), Shibuya (2011) |
| Risk communication | Policies and practices for communicating about public health emergencies with the community. | 27 | Andrus (2017), Ardagh (2012), Beard (2003), Bracha (2006), Chaple (2017), Cleary (2010), Dhariwal (2004), Dickmann (2016), Elston (2015), Felland (2008), Fukuma (2017), Gizelis (2017), Gostin (2015), Ivers (2011), Kruk (2015), Lapão (2015), Meyer (2017), Moore (2015), Pouraghaei (2017), Quaglio (2016), Regmi (2015), Rockefeller (2016), Rozeman (2006), World Health Organization (2017), Sesay (2017), Shultz (2017), Varma (2017) |
| Surge capacity | Policies and practices necessary to accommodate a surge of patients during a public health emergency. | 25 | Balfour (2016), Bracha (2006), Cancedda (2016), Chaple (2017), Elliott (2010), Elston (2017), Felland (2008), Fukuma (2017), Ivers (2011), Kelen (2009), Kim (2009), Kluge (2017), Kruk (2017), Olu (2017), Pouraghaei (2017), Rozeman (2006), Schenk (2017), Senga (2016), Shultz (2017), Singh (2017), Subhash (2016), Therrien (2016), Varma (2017), Verni (2012), Zhong (2014) |
| Workforce | The workforce (and associated capabilities) needed to respond to a public health emergency. | 55 | Akbari (2005), Ammar (2016), Andrus (2017), Ardagh (2012), Barasa (2017), Barden-O’Fallon (2015), Baumann (2006), Budy (2015), Cancedda (2016), Copper (2016), DeBiasi (2015), Dhariwal (2004), Elliott (2010), Elston (2015), Felland (2008), Fitter (2017), Fakuma (2017), Gizelis (2017), Gostin (2015), Hanefeld (2018), Harries (2010), Jones (2017), Kao (2017), Kennedy (2016), Schwanke Khilji (2013), Kim (2009), Kluge (2017), Kruk (2015), Kruk (2017), Lewis (2017), Liu (2004), Lohman (2016), Lurie (2004), McPake (2015), Meyer (2017), Van Minh (2014), Moore (2015), Mussah (2017), Nyenswah (2017), Pouraghaei (2017), Raven (2018), Reece (2017), Rozeman (2006), Sesay (2017), Shibuya (2011), Shoman (2017), Shultz (2017), Singh (2017), Therrien (2016), Tomori (20150, Tran (2015), Varma (2017), Verni (2012), Wurie (2016), Zhong (2014) |

**Appendix B: Citations**

1. Akbari ME, Asadi Lari M, Montazeri A, Aflatunian MR, Farshad AA. Evaluation of Health System Responsiveness to the 2003 Bam, Iran, Earthquake. Earthquake Spectra. 2005 Dec 1;21(S1):469–74.

2. Ammar W, Kdouh O, Hammoud R, Hamadeh R, Harb H, Ammar Z, et al. Health system resilience: Lebanon and the Syrian refugee crisis. J Glob Health [Internet]. 2016;6(2). Available from: https://www.ncbi.nlm.nih.gov/pmc/articles/PMC5234495/

3. Andrus JK, Cochi SL, Cooper LZ, Klein JD. Combining Global Elimination Of Measles And Rubella With Strengthening Of Health Systems In Developing Countries. Health Affairs. 2016;35(2).

4. Ardagh MW, Richardson SK, Robinson V, Than M, Gee P, Henderson S, et al. The initial health-system response to the earthquake in Christchurch, New Zealand, in February, 2011. The Lancet. 2012 Jun 2;379(9831):2109–15.

5. Ardalan A, Masoomi G, Goya M, Ghaffari M, Miadfar J, Sarvar M, et al. Disaster Health Management: Iran’s Progress and Challenges. Iran J Public Health. 2009;38(Supplement 1):93–7.

6. Baers JH, Wiley K, Davies JM, Caird JK, Hallihan G, Conly J. A Health System’s Preparedness for the “Next Ebola.” Ergonomics in Design. 2018 Jan 1;26(1):24–8.

7. Balfour E, Stallone R, Castagnaro J, Poczter H, Schron D, Martone J, et al. Strengths of the Northwell Health Laboratory Service Line: Maintaining Performance During Threatened Interruptions in Service. Acad Pathol. 2016 Dec;3:2374289516650961.

8. Barasa EW, Cloete K, Gilson L. From bouncing back, to nurturing emergence: reframing the concept of resilience in health systems strengthening. Health Policy Plan. 2017 Nov 1;32(suppl_3):iii91-iii94.

9. Barden-O’Fallon J, Barry MA, Brodish P, Hazerjian J. Rapid Assessment of Ebola-Related Implications for Reproductive, Maternal, Newborn and Child Health Service Delivery and Utilization in Guinea. PLoS Curr [Internet]. 2015 Aug 4 [cited 2018 Apr 24]; Available from: http://currents.plos.org/outbreaks/article/rapid-assessment-of-ebola-related-implications-for-reproductive-maternal-newborn-and-child-health-service-delivery-and-utilization-in-guinea/

10. Baumann AO, Blythe JM, Underwood JM. Surge capacity and casualization: Human resource issues in the post-SARS health system. Can J Public Health. 2006 Jun;97(3):230–2.

11. Beard L, Clark C. SARS: a health system’s perspective. Hosp Q. 2003;6(4):55–8, 4.

12. Blanchet K, Nam SL, Ramalingam B, Pozo-Martin F. Governance and Capacity to Manage Resilience of Health Systems: Towards a New Conceptual Framework. Int J Health Policy Manag. 2017 Apr 4;6(8):431–5.

13. Bolkan HA, Bash-Taqi DA, Samai M, Gerdin M, von Schreeb J. Ebola and indirect effects on health service function in sierra leone. PLoS Curr. 2014 Dec 19;6.

14. Bracha HS, Burkle FM. Utility of fear severity and individual resilience scoring as a surge capacity, triage management tool during large-scale, bio-event disasters. Prehosp Disaster Med. 2006 Oct;21(5):290-296-298.

15. Budy FCT. Policy Options for Addressing Health System and Human Resources for Health Crisis in Liberia Post-Ebola Epidemic. Int J MCH AIDS. 2015;4(2):1–7.

16. Campbell A. The SARS Commission Interim Report: SARS and Public Health in Ontario. Biosecurity and Bioterrorism: Biodefense Strategy, Practice, and Science. 2004 Jun 1;2(2):118–26.

17. Cancedda C, Davis SM, Dierberg KL, Lascher J, Kelly JD, Barrie MB, et al. Strengthening Health Systems While Responding to a Health Crisis: Lessons Learned by a Nongovernmental Organization During the Ebola Virus Disease Epidemic in Sierra Leone. J Infect Dis. 2016 Oct 15;214(suppl 3):S153–63.

18. Caulker VML, Mishra S, van Griensven J, Moosa A, Najjemba R, Shringarpure K, et al. Life goes on: the resilience of maternal primary care during the Ebola outbreak in rural Sierra Leone. Public Health Action. 2017 Jun 21;7(Suppl 1):S40–6.

19. Chaple EB, Mercer MA. The Cuban Response to the Ebola Epidemic in West Africa: Lessons in Solidarity. Int J Health Serv. 2017;47(1):134–49.

20. Chi GC, Williams M, Chandra A, Plough A, Eisenman D. Partnerships for community resilience: perspectives from the Los Angeles County Community Disaster Resilience project. Public Health. 2015 Sep;129(9):1297–300.

21. Cleary V, Balasegaram S, McCloskey B, Keeling D, Turbitt D. Pandemic (H1N1) 2009: setting up a multi-agency regional response centre--a toolkit for other public health emergencies. J Bus Contin Emer Plan. 2010 Mar;4(2):154–64.

22. Cooper C, Fisher D, Gupta N, MaCauley R, Pessoa-Silva CL. Infection prevention and control of the Ebola outbreak in Liberia, 2014–2015: key challenges and successes. BMC Med [Internet]. 2016 Jan 5;14. Available from: https://www.ncbi.nlm.nih.gov/pmc/articles/PMC4702360/

23. DeBiasi RL, Song X, Cato K, Floyd T, Talley L, Gorman K, et al. Preparedness, Evaluation, and Care of Pediatric Patients Under Investigation for Ebola Virus Disease: Experience from a Pediatric Designated Care Facility. J Pediatric Infect Dis Soc. 2016 Mar;5(1):68–75.

24. Dhanoa T, Fuller H, Herechuk B, Trowbridge S, Raab V, MacDonald AM, et al. Response to a Serious Flood: The St Joseph’s Healthcare Experience. Healthc Q. 2014;17(4):58–62.

25. Dhariwal H-RS. Disaster Resilience of the Vancouver Health Care System to Pandemic Influenza [Internet]. The University of Victoria; 2004. Available from: file:///Users/dianemeyer/Downloads/ubc_2009_spring_dhariwal_har-rajandeep.pdf

26. Dickmann P, Apfel F, Gottschalk R. Risk communication and generic preparedness: from agent-based to action-based planning - a conceptual framework. British Journal of Medicine and Medical Research. 2016 Feb 14;13:1–5.

27. Elliott A, Rehfisch N. Mortuary provision in emergencies causing mass fatalities. J Bus Contin Emer Plan. 2011 Feb;5(1):430–9.

28. Elmahdawy M, Elsisi GH, Carapinha J, Lamorde M, Habib A, Agyie-Baffour P, et al. Ebola Virus Epidemic in West Africa: Global Health Economic Challenges, Lessons Learned, and Policy Recommendations. Value Health Reg Issues. 2017 Sep;13:67–70.

29. Elston JWT, Cartwright C, Ndumbi P, Wright J. The health impact of the 2014–15 Ebola outbreak. Public Health. 2017 Feb 1;143:60–70.

30. Elston JWT, Moosa AJ, Moses F, Walker G, Dotta N, Waldman RJ, et al. Impact of the Ebola outbreak on health systems and population health in Sierra Leone. J Public Health (Oxf). 2016 Dec 2;38(4):673–8.

31. Felland LE, Katz A, Liebhaber A, Cohen GR. Developing health system surge capacity: community efforts in jeopardy. Res Brief. 2008 Jun;(5):1–8.

32. Fitter DL, Delson DB, Guillaume FD, Schaad AW, Moffett DB, Poncelet J-L, et al. Applying a New Framework for Public Health Systems Recovery following Emergencies and Disasters: The Example of Haiti following a Major Earthquake and Cholera Outbreak. Am J Trop Med Hyg. 2017 Oct;97(4_Suppl):4–11.

33. Fukuma S, Ahmed S, Goto R, Inui TS, Atun R, Fukuhara S. Fukushima after the Great East Japan Earthquake: lessons for developing responsive and resilient health systems. J Glob Health. 2017 Jun;7(1):10501.

34. Garibaldi BT, Kelen GD, Brower RG, Bova G, Ernst N, Reimers M, et al. The Creation of a Biocontainment Unit at a Tertiary Care Hospital. The Johns Hopkins Medicine Experience. Ann Am Thorac Soc. 2016;13(5):600–8.

35. Gauld R. “Exposing the cracks”: Severe Acute Respiratory Syndrome and the Hong Kong health system. J of Health Org and Mgt. 2005 Apr 1;19(2):106–19.

36. Gizelis T-I, Karim S, Østby G, Urdal H. Maternal Health Care in the Time of Ebola: A Mixed-Method Exploration of the Impact of the Epidemic on Delivery Services in Monrovia. World Development. 2017 Oct 1;98:169–78.

37. Gostin LO, Friedman EA. A retrospective and prospective analysis of the west African Ebola virus disease epidemic: robust national health systems at the foundation and an empowered WHO at the apex. The Lancet. 2015 May 9;385(9980):1902–9.

38. Griekspoor A, Spiegel P, Aldis W, Harvey P. The Health Sector Gap in the Southern Africa Crisis in 2002/2003. Disasters. 2004;28(4):388–404.

39. Hafner T, Walkowiak H, Lee D, Aboagye-Nyame F. Defining pharmaceutical systems strengthening: concepts to enable measurement. Health Policy Plan. 2017 May 1;32(4):572–84.

40. Hanefeld J, Mayhew S, Legido-Quigley H, Martineau F, Karanikolos M, Blanchet K, et al. Towards an understanding of resilience: responding to health systems shocks. Health Policy Plan. 2018 Apr 1;33(3):355–67.

41. Harries AD, Zachariah R, Tayler-Smith K, Schouten EJ, Chimbwandira F, Van Damme W, et al. Keeping health facilities safe: one way of strengthening the interaction between disease-specific programmes and health systems. Trop Med Int Health. 2010 Dec;15(12):1407–12.

42. Hartmann EH, Creel N, Lepard J, Maxwell RA. Mass casualty following unprecedented tornadic events in the Southeast: natural disaster outcomes at a Level I trauma center. Am Surg. 2012 Jul;78(7):770–3.

43. Institute of Medicine (US). Barriers to Integrating Crisis Standards of Care Principles into International Disaster Response Plans: Workshop Summary [Internet]. Washington (DC): National Academies Press (US); 2012. (The National Academies Collection: Reports funded by National Institutes of Health). Available from: http://www.ncbi.nlm.nih.gov/books/NBK91501/

44. Isakov A, Gibbs S, Lowe J, Jamison A, Swansiger R. Transport and Management of Patients With Confirmed or Suspected Ebola Virus Disease. Ann Emerg Med. 2015 Sep;66(3):297–305.

45. Ivers LC. Strengthening the health system while investing in Haiti. Am J Public Health. 2011 Jun;101(6):970–1.

46. Jones S, Sam B, Bull F, Pieh SB, Lambert J, Mgawadere F, et al. “Even when you are afraid, you stay”: Provision of maternity care during the Ebola virus epidemic: A qualitative study. Midwifery. 2017 Sep;52:19–26.

47. Jones SA, Gopalakrishnan S, Ameh CA, White S, van den Broek NR. “Women and babies are dying but not of Ebola”: the effect of the Ebola virus epidemic on the availability, uptake and outcomes of maternal and newborn health services in Sierra Leone. BMJ Glob Health. 2016;1(3):e000065.

48. Kao H-Y, Ko H-Y, Guo P, Chen C-H, Chou S-M. Taiwan’s Experience in Hospital Preparedness and Response for Emerging Infectious Diseases. Health Secur. 2017 Apr;15(2):175–84.

49. Karanikolos M, Heino P, McKee M, Stuckler D, Legido-Quigley H. Effects of the Global Financial Crisis on Health in High-Income Oecd Countries: A Narrative Review. Int J Health Serv. 2016;46(2):208–40.

50. Kearns RD, Holmes JH, Skarote MB, Cairns CB, Strickland SC, Smith HG, et al. Disasters; the 2010 Haitian earthquake and the evacuation of burn victims to US burn centers. Burns. 2014 Sep;40(6):1121–32.

51. Kelen GD, McCarthy ML, Kraus CK, Ding R, Hsu EB, Li G, et al. Creation of surge capacity by early discharge of hospitalized patients at low risk for untoward events. Disaster Med Public Health Prep. 2009 Jun;3(2 Suppl):S10-16.

52. Kennedy SB, Dogba JB, Wasunna CL, Sahr P, Eastman CB, Bolay FK, et al. Pre-Ebola virus disease laboratory system and related challenges in Liberia. Afr J Lab Med. 2016;5(3):508.

53. Kim CS, Pile JC, Lozon MM, Wilkerson WM, Wright CM, Cinti S. Role of hospitalists in an offsite alternate care center (ACC) for pandemic flu. J Hosp Med. 2009 Nov;4(9):546–9.

54. Kingham TP, Kamara TB, Cherian MN, Gosselin RA, Simkins M, Meissner C, et al. Quantifying surgical capacity in Sierra Leone: a guide for improving surgical care. Arch Surg. 2009 Feb;144(2):122–127; discussion 128.

55. Kluge H, Martín-Moreno JM, Emiroglu N, Rodier G, Kelley E, Vujnovic M, et al. Strengthening global health security by embedding the International Health Regulations requirements into national health systems. BMJ Global Health. 2018 Jan 1;3(Suppl 1):e000656.

56. Kreisberg D, Thomas DSK, Valley M, Newell S, Janes E, Little C. Vulnerable Populations in Hospital and Health Care Emergency Preparedness Planning: A Comprehensive Framework for Inclusion. Prehosp Disaster Med. 2016 Apr;31(2):211–9.

57. Kruk ME, Ling EJ, Bitton A, Cammett M, Cavanaugh K, Chopra M, et al. Building resilient health systems: a proposal for a resilience index. BMJ. 2017 23;357:j2323.

58. Kruk ME, Myers M, Varpilah ST, Dahn BT. What is a resilient health system? Lessons from Ebola. The Lancet. 2015 May 9;385(9980):1910–2.

59. Lapão LV, Silva A, Pereira N, Vasconcelos P, Conceição C. Ebola impact on African health systems entails a quest for more international and local resilience: the case of African Portuguese speaking countries. Pan Afr Med J. 2015;22 Suppl 1:15.

60. Lewis JD, Enfield KB, Perl TM, Sifri CD. Preparedness planning and care of patients under investigation for or with Ebola virus disease: A survey of physicians in North America. Am J Infect Control. 2017 Jan 1;45(1):65–8.

61. Li W, Jalloh MF, Bunnell R, Aki-Sawyerr Y, Conteh L, Sengeh P, et al. Public Confidence in the Health Care System 1 Year After the Start of the Ebola Virus Disease Outbreak - Sierra Leone, July 2015. MMWR Morb Mortal Wkly Rep. 2016 Jun 3;65(21):538–42.

62. Lien C, Raimo J, Abramowitz J, Khanijo S, Kritharis A, Mason C, et al. Community healthcare delivery post-Hurricane Sandy: lessons from a mobile health unit. J Community Health. 2014 Jun;39(3):599–605.

63. Ling EJ, Larson E, Macauley RJ, Kodl Y, VanDeBogert B, Baawo S, et al. Beyond the crisis: did the Ebola epidemic improve resilience of Liberia’s health system? Health Policy Plan. 2017 Nov 1;32(suppl_3):iii40-iii47.

64. Liu Y. China’s public health-care system: facing the challenges. Bull World Health Organ. 2004 Jul;82(7):532–8.

65. Lohman N, Hagopian A, Luboga SA, Stover B, Lim T, Makumbi F, et al. District Health Officer Perceptions of PEPFAR’s Influence on the Health System in Uganda, 2005-2011. Int J Health Policy Manag. 2016 Jul 26;6(2):83–95.

66. Loubet P, Mabileau G, Baysah M, Nuta C, Taylor M, Jusu H, et al. Likely effect of the 2014 Ebola epidemic on HIV care in Liberia. AIDS. 2015 Nov;29(17):2347–51.

67. Lurie N, Valdez RB, Wasserman J, Stoto MA, Myers S, Molander RC, et al. Public Health Preparedness in California: Lessons Learned from Seven Health Jurisdictions [Internet]. RAND Corporation; 2004 [cited 2019 Apr 15]. Available from: https://www.rand.org/pubs/technical_reports/TR181.html

68. Macintyre K, Hotchkiss DR. Referral revisited: community financing schemes and emergency transport in rural Africa. Soc Sci Med. 1999 Dec;49(11):1473–87.

69. Martineau FP. People-centred health systems: building more resilient health systems in the wake of the Ebola crisis. Int Health. 2016 Sep 1;8(5):307–9.

70. McMahon SA, Ho LS, Scott K, Brown H, Miller L, Ratnayake R, et al. “We and the nurses are now working with one voice”: How community leaders and health committee members describe their role in Sierra Leone’s Ebola response. BMC Health Serv Res. 2017 18;17(1):495.

71. McPake B, Witter S, Ssali S, Wurie H, Namakula J, Ssengooba F. Ebola in the context of conflict affected states and health systems: case studies of Northern Uganda and Sierra Leone. Conflict and Health. 2015 Aug 8;9:23.

72. Mehta S. Disaster and mass casualty management in a hospital: How well are we prepared? Journal of Postgraduate Medicine. 2006 Apr 1;52(2):89.

73. Meyer D, Kirk Sell T, Schoch-Spana M, Shearer MP, Chandler H, Thomas E, et al. Lessons from the domestic Ebola response: Improving health care system resilience to high consequence infectious diseases. Am J Infect Control. 2017 Dec 15;

74. Moore BL, Geller RJ, Clark C. Hospital preparedness for chemical and radiological disasters. Emerg Med Clin North Am. 2015 Feb;33(1):37–49.

75. Mussah VG, Mapleh L, Ade S, Harries AD, Bhat P, Kateh F, et al. Performance-based financing contributes to the resilience of health services affected by the Liberian Ebola outbreak. Public Health Action. 2017 Jun 21;7(Suppl 1):S100–5.

76. Mutale W, Ayles H, Bond V, Chintu N, Chilengi R, Mwanamwenge MT, et al. Application of systems thinking: 12-month postintervention evaluation of a complex health system intervention in Zambia: the case of the BHOMA. J Eval Clin Pract. 2017 Apr;23(2):439–52.

77. Mutale W, Balabanova D, Chintu N, Mwanamwenge MT, Ayles H. Application of system thinking concepts in health system strengthening in low-income settings: a proposed conceptual framework for the evaluation of a complex health system intervention: the case of the BHOMA intervention in Zambia. J Eval Clin Pract. 2016 Feb;22(1):112–21.

78. Nyenswah T. Reflections on Leadership and Governance from the Incident Manager of Liberia’s Ebola Response. Health Secur. 2017 Aug;15(4):445–9.

79. Oliveira C, Russo G. Vertical interventions and system effects; have we learned anything from past experiences? Pan Afr Med J. 2015;21:262.

80. Olu O, Petu A, Ovberedjo M, Muhongerwa D. South-South cooperation as a mechanism to strengthen public health services in Africa: experiences, challenges and a call for concerted action. Pan Afr Med J [Internet]. 2017 Sep 15;28. Available from: https://www.ncbi.nlm.nih.gov/pmc/articles/PMC5687874/

81. Olu O. Resilient Health System As Conceptual Framework for Strengthening Public Health Disaster Risk Management: An African Viewpoint. Front Public Health [Internet]. 2017 Sep 28;5. Available from: https://www.ncbi.nlm.nih.gov/pmc/articles/PMC5625001/

82. Omoleke SA, Mohammed I, Saidu Y. Ebola Viral Disease in West Africa: A Threat to Global Health, Economy and Political Stability. J Public Health Africa [Internet]. 2016 Aug 17;7(1). Available from: https://www.ncbi.nlm.nih.gov/pmc/articles/PMC5349256/

83. Orenstein WA, Seib K. Beyond vertical and horizontal programs: a diagonal approach to building national immunization programs through measles elimination. Expert Rev Vaccines. 2016;15(7):791–3.

84. Pathmanathan I, O’Connor KA, Adams ML, Rao CY, Kilmarx PH, Park BJ, et al. Rapid assessment of Ebola infection prevention and control needs--six districts, Sierra Leone, October 2014. MMWR Morb Mortal Wkly Rep. 2014 Dec 12;63(49):1172–4.

85. Paudel YR, Dariang M, Keeling SJ, Mehata S. Addressing the needs of people with disability in Nepal: The urgent need. Disabil Health J. 2016 Apr;9(2):186–8.

86. Peters D. The Ebola epidemic in Liberia: the role of communities and local leadership in overcoming catastrophe and building health system resilienceDavid Peters. Eur J Public Health [Internet]. 2017 Nov 1 [cited 2019 Apr 15];27(suppl_3). Available from: https://academic.oup.com/eurpub/article/27/suppl_3/ckx187.588/4556554

87. Pouraghaei M, Jannati A, Moharamzadeh P, Ghaffarzad A, Heshmati Far M, Babaie J. Challenges of Hospital Response to the Twin Earthquakes of August 21, 2012, in East Azerbaijan, Iran. Disaster medicine and public health preparedness. 2017 Jan 9;11:1–9.

88. Quaglio G, Pizzol D, Bome D, Kebbie A, Bangura Z, Massaquoi V, et al. Maintaining Maternal and Child Health Services During the Ebola Outbreak: Experience from Pujehun, Sierra Leone. PLoS Curr [Internet]. 2016 Jun 2 [cited 2019 Apr 15]; Available from: index.html%3Fp=66380.html

89. Raven J, Baral S, Wurie H, Witter S, Samai M, Paudel P, et al. What adaptation to research is needed following crises: a comparative, qualitative study of the health workforce in Sierra Leone and Nepal. Health Res Policy Syst. 2018 Feb 7;16(1):6.

90. Read DJ, Holian A, Moller C-C, Poutawera V. Surgical workload of a foreign medical team after Typhoon Haiyan. ANZ J Surg. 2016 May;86(5):361–5.

91. Rebmann T, McPhee K, Osborne L, Gillen DP, Haas GA. Best Practices for Healthcare Facility and Regional Stockpile Maintenance and Sustainment: A Literature Review. Health Secur. 2017 Aug;15(4):409–17.

92. Reece S, Brown CS, Dunning J, Chand MA, Zambon MC, Jacobs M. The UK’s multidisciplinary response to an Ebola epidemic. Clin Med (Lond). 2017 Jul;17(4):332–7.

93. Regmi K, Gilbert R, Thunhurst C. How can health systems be strengthened to control and prevent an Ebola outbreak? A narrative review. Infect Ecol Epidemiol. 2015;5:28877.

94. Reinhardt JD, Li J, Gosney J, Rathore FA, Haig AJ, Marx M, et al. Disability and health-related rehabilitation in international disaster relief. Glob Health Action. 2011;4:7191.

95. Rozeman PA, Mayeaux EJ. Hurricanes Katrina and Rita: evacuee healthcare efforts remote from hurricane affected areas. South Med J. 2006 Dec;99(12):1329–33.

96. Saghafi Nia M. Survey of the bam earthquake survivors’ opinions on medical and health system services. Prehosp Disaster Med. 2008 Aug;23(4):382.

97. Saurabh S, Prateek S. Role of contact tracing in containing the 2014 Ebola outbreak: a review. Afr Health Sci. 2017 Mar;17(1):225–36.

98. Schenk WC, Bui T. Sri Lanka’s post-tsunami health system recovery: a qualitative analysis of physician perspectives. Int Health. 2018 01;10(1):20–6.

99. Schwanke Khilji SU, Rudge JW, Drake T, Chavez I, Borin K, Touch S, et al. Distribution of selected healthcare resources for influenza pandemic response in Cambodia. International Journal for Equity in Health. 2013 Oct 4;12:82.

100. Senga M, Pringle K, Ramsay A, Brett-Major DM, Fowler RA, French I, et al. Factors Underlying Ebola Virus Infection Among Health Workers, Kenema, Sierra Leone, 2014–2015. Clin Infect Dis. 2016 Aug 15;63(4):454–9.

101. Sesay T, Denisiuk O, Shringarpure KK, Wurie BS, George P, Sesay MI, et al. Paediatric care in relation to the 2014–2015 Ebola outbreak and general reporting of deaths in Sierra Leone. Public Health Action. 2017 Jun 21;7(Suppl 1):S34–9.

102. Setiawan GP, Viora E. Disaster mental health preparedness plan in Indonesia. Int Rev Psychiatry. 2006 Dec;18(6):563–6.

103. Shamian J, Petch T, Lilius F, Shainblum E, Talosi R. What’s the plan? The unique challenges facing the home and community care sector in preparing for a pandemic. Healthc Pap. 2007;8(1):38-43-55.

104. Shannon FQ, Horace-Kwemi E, Najjemba R, Owiti P, Edwards J, Shringarpure K, et al. Effects of the 2014 Ebola outbreak on antenatal care and delivery outcomes in Liberia: a nationwide analysis. Public Health Action. 2017 Jun 21;7(Suppl 1):S88–93.

105. Shibuya K, Hashimoto H, Ikegami N, Nishi A, Tanimoto T, Miyata H, et al. Future of Japan’s system of good health at low cost with equity: beyond universal coverage. Lancet. 2011 Oct 1;378(9798):1265–73.

106. Shoaf K, Shoaf K. Organizing the health sector for response to disasters. Ciência & Saúde Coletiva. 2014 Sep;19(9):3705–15.

107. Shoman H, Karafillakis E, Rawaf S. The link between the West African Ebola outbreak and health systems in Guinea, Liberia and Sierra Leone: a systematic review. Global Health [Internet]. 2017 Jan 4;13. Available from: https://www.ncbi.nlm.nih.gov/pmc/articles/PMC5210305/

108. Shultz JM, Cooper JL, Baingana F, Oquendo MA, Espinel Z, Althouse BM, et al. The Role of Fear-Related Behaviors in the 2013-2016 West Africa Ebola Virus Disease Outbreak. Curr Psychiatry Rep. 2016 Nov;18(11):104.

109. Siekmans K, Sohani S, Boima T, Koffa F, Basil L, Laaziz S. Community-based health care is an essential component of a resilient health system: evidence from Ebola outbreak in Liberia. BMC Public Health [Internet]. 2017 Jan 17;17. Available from: https://www.ncbi.nlm.nih.gov/pmc/articles/PMC5240441/

110. Simonetti A, Ezzeldin H, Walderhaug M, Anderson SA, Forshee RA. An Inter-regional US Blood Supply Simulation Model to Evaluate Blood Availability to Support Planning for Emergency Preparedness and Medical Countermeasures. Disaster Medicine and Public Health Preparedness. 2018 Apr;12(2):201–10.

111. Singh SR, Coker R, Vrijhoef HJ-M, Leo YS, Chow A, Lim PL, et al. Mapping infectious disease hospital surge threats to lessons learnt in Singapore: a systems analysis and development of a framework to inform how to DECIDE on planning and response strategies. BMC Health Serv Res. 2017 Sep 4;17(1):622.

112. Subhash SS, Baracco G, Miller SL, Eagan A, Radonovich LJ. Estimation of Needed Isolation Capacity for an Airborne Influenza Pandemic. Health Secur. 2016 Aug;14(4):258–63.

113. Tambo E, Chengho CF, Ugwu CE, Wurie I, Jonhson JK, Ngogang JY. Rebuilding transformation strategies in post-Ebola epidemics in Africa. Infect Dis Poverty. 2017 May 10;6(1):71.

114. Therrien M-C, Normandin J-M, Denis J-L. Bridging complexity theory and resilience to develop surge capacity in health systems. J of Health Org and Mgt. 2017 Feb 3;31(1):96–109.

115. Tomori O. Will Africa’s future epidemic ride on forgotten lessons from the Ebola epidemic? BMC Medicine. 2015 May 14;13(1):116.

116. Tran TM, Saint-Fort M, Jose M-D, Henrys JH, Pierre Pierre JB, Cherian MN, et al. Estimation of Surgery Capacity in Haiti: Nationwide Survey of Hospitals. World J Surg. 2015 Sep;39(9):2182–90.

117. Van Minh H, Tuan Anh T, Rocklöv J, Bao Giang K, Trang LQ, Sahlen K-G, et al. Primary healthcare system capacities for responding to storm and flood-related health problems: a case study from a rural district in central Vietnam. Glob Health Action [Internet]. 2014 Dec 8;7. Available from: https://www.ncbi.nlm.nih.gov/pmc/articles/PMC4265642/

118. Varma JK, Prezant DJ, Wilson R, Quinn C, Asaeda G, Cagliuso NV, et al. Preparing the Health System to Respond to Ebola Virus Disease in New York City, 2014. Disaster Med Public Health Prep. 2017 Jun;11(3):370–4.

119. Veenema TG, Thornton CP, Lavin RP, Bender AK, Seal S, Corley A. Climate Change-Related Water Disasters’ Impact on Population Health. J Nurs Scholarsh. 2017;49(6):625–34.

120. Verni C. A hospital system’s response to a hurricane offers lessons, including the need for mandatory interfacility drills. Health Aff (Millwood). 2012 Aug;31(8):1814–21.

121. Wurie HR, Samai M, Witter S. Retention of health workers in rural Sierra Leone: findings from life histories. Human Resources for Health. 2016 Feb 1;14:3.

122. Yakubu A, Folayan MO, Sani-Gwarzo N, Nguku P, Peterson K, Brown B. The Ebola outbreak in Western Africa: ethical obligations for care. Journal of Medical Ethics. 2014 Sep 9;medethics-2014-102434.

123. Yantao X. Assessment of hospital emergency management in the Beijing area. Prehosp Disaster Med. 2011 Jun;26(3):180–3.

124. Zhang X, Bloom G, Xu X, Chen L, Liang X, Wolcott SJ. Advancing the application of systems thinking in health: managing rural China health system development in complex and dynamic contexts. Health Research Policy and Systems. 2014 Aug 26;12(1):44.

125. Zhong S, Clark M, Hou X-Y, Zang Y, FitzGerald G. Progress and challenges of disaster health management in China: a scoping review. Glob Health Action. 2014;7:24986.

126. Hospital Safety Index: Evaluation Forms for Safe Hospitals [Internet]. Pan American Health Organization; 2008 [cited 2019 Apr 15]. Available from: https://www.paho.org/disasters/index.php?option=com_content&view=article&id=964:safety-index&Itemid=912&lang=en

127. Leadership During a Pandemic: What Your Municipality Can Do (Tool 19: Recovery and Resilience) [Internet]. USAID; 2011. Available from: https://www.paho.org/disasters/index.php?option=com_docman&view=download&category_slug=tools&alias=546-pandinflu-leadershipduring-tool-19&Itemid=1179&lang=en

128. Post-Disaster Needs Assessment Guidelines Volume B: Health [Internet]. European Union, UN Development Group, World Bank; 2014 Aug. Available from: https://www.gfdrr.org/sites/gfdrr/files/WB_UNDP_PDNA_Health_SP_FINAL.pdf

129. Hospitals prepare plans, drill staff to ensure that potential Ebola patients are identified, isolated, and managed safely. ED Manag. 2014 Dec;26(12):138–41.

130. Effective Public Health Communication in an Interconnected World: Enhancing Resilience to Health Crises [Internet]. The Rockefeller Foundation; 2016 Apr. Available from: https://www.rockefellerfoundation.org/report/effective-public-health-communication-in-an-interconnected-world-enhancing-resilience-to-health-crises/

131. Recovery toolkit: Supporting countries to achieve health service resilience [Internet]. World Health Organization; 2016 May. Available from: https://www.who.int/csr/resources/publications/ebola/recovery-toolkit/en/

132. Strengthening resilience: a priority shared by Health 2020 and the Sustainable Development Goals [Internet]. World Health Organization; 2017. Available from: http://www.euro.who.int/__data/assets/pdf_file/0005/351284/resilience-report-20171004-h1635.pdf
