# Supplementary material for: What makes health systems resilient against infectious disease outbreaks and natural hazards? Results from a scoping review
Source: BMC Public Health. 2019 Oct 17;19:1310. doi: 10.1186/s12889-019-7707-z (PMC6798426; doi:10.1186/s12889-019-7707-z)
Supplement: Supplementary file 3 — Additional file 3: Figure S1. Selection of Sources of Evidence. [file 12889_2019_7707_MOESM3_ESM.docx]

Final coding sample (132)

Full document review (158)

Review of references (8)

Documents from organizational websites (9)

Eliminated after whole article review (26)

Eliminated after abstract review (283)

Eliminated after title review (684)

Duplicates eliminated (789)

Abstract review (424)

Title review (1108)

1897 articles identified in initial search

Final list of key evidence (77)
